# Analytical Evaluation and Antioxidant Properties of Some Secondary Metabolites in Northern Italian Mono- and Multi-Varietal Extra Virgin Olive Oils (EVOOs) from Early and Late Harvested Olives

**DOI:** 10.3390/ijms18040797

**Published:** 2017-04-11

**Authors:** Domenico Trombetta, Antonella Smeriglio, Daniele Marcoccia, Salvatore Vincenzo Giofrè, Giovanni Toscano, Fabio Mazzotti, Angelo Giovanazzi, Stefano Lorenzetti

**Affiliations:** 1 Department of Chemical, Biological, Pharmaceutical and Environmental Sciences, University of Messina, viale F. Stagno d’Alcontres 31, 98100 Messina, Italy; dtrombetta@unime.it (D.T.); asmeriglio@unime.it (A.S.); sgiofre@unime.it (S.V.G.); gtoscano@unime.it (G.T.); 2 Istituto Superiore di Sanità, Department of Food Safety and Veterinary Public Health, Unit of Food and Veterinary Toxicology, viale Regina Elena 299, 00161 Rome, Italy; daniele430@alice.it; 3 Department of Chemistry and Chemical Technologies, University of Calabria, via P. Bucci, Cubo 12/C, 87036 Arcavacata di Rende (Cosenza), Italy; fmazzotti@unical.it; 4 Rovereto town council, 38068 Rovereto (Trento), Italy; angelo.giovanazzi@gmail.com

**Keywords:** extra virgin olive oil (EVOO), functional food, polyphenols, α-tocopherol, antioxidant activity, harvest time

## Abstract

The antioxidant activity and the phenolic and α-tocopherol content of 10 Northern Italian mono- and multi-varietal extra virgin olive oils (EVOOs), after early and late olive harvests, was analyzed. A hierarchical cluster analysis was used to evaluate sample similarity. Secoiridoids (SIDs), lignans and flavonoids were the most abundant phenolic compounds identified. The organic Casaliva (among mono-cultivar) and the organic multi-varietal (among blended oils) EVOOs had the higher total phenol content both in early (263.13 and 326.19 mg/kg, respectively) and late harvest (241.88 and 292.34 mg/kg, respectively) conditions. In comparison to late harvest EVOOs, early harvest EVOOs, in particular the organic mono-cultivar Casaliva, showed both higher antioxidant capacity (up to 1285.97 Oxygen Radicals Absorbance Capacity/ORAC units), probably due to the higher SID fraction (54% vs. 40%), and higher α-tocopherol content (up to 280.67 mg/kg). Overall, these results suggest that SIDs and α-tocopherol mainly contribute to antioxidant properties of the studied EVOOs. In light of this, the authors conclude that early harvest, organic mono-cultivar Casaliva EVOO represents the most interesting candidate to explicate healthy effects ascribed to these functional constituents, particularly regarding oxidative stress-related pathologies.

## 1. Introduction

Mediterranean lifestyle includes the dietary intake of several food-derived chemicals that are thought to confer protective health effects, including the consumption of 25–30 g/day of extra virgin olive oil (EVOO) as the principal source of fats [1,2]. On the other hand, a recent meta- analysis of EFSA (European Food Safety Authority) suggested a consumption lower than 20 g olive oil per day but containing at least 5 mg hydroxytyrosol and derivatives [3].

EVOO is produced purely by mechanical pressure and without any chemical processing and is particularly rich in natural antioxidants that prevent the insurgence of several pathologies. The nutritional quality and the health benefits of EVOO are attributed both to its balanced fatty acid composition (high content of oleic acid and optimal ratio between ω-3 and ω-6 fatty acids) and to a significant amount of minor components with antioxidant properties in the unsaponifiable fraction, such as tocopherols and phenolic compounds [4,5]. These latter compounds have been shown, in vivo and in vitro, to possess antimicrobial, antioxidant, anti-allergic, anti-inflammatory, anti-carcinogenic, and antiviral properties [2]. They are also important to confer aromatic flavor notes [6] and, in comparison with other vegetable oils, to maintain a significant stability in terms of resistance against self-oxidation and thermal oxidation processes [7]. Tocopherols are present in olive oil in the forms α, β, γ, and δ, with 90% being found in the most biologically active α form, vitamin E [5]. Experimental evidence has demonstrated that the phenolic compounds present in EVOO possess numerous biological properties and are, at least partly, responsible for the beneficial effects arising from the consumption of EVOO [8,9,10].

To date, a great deal of variability in the amount and composition of phenolic compounds identified in EVOOs has been determined ranging from 0.02 to 600 mg/kg [2]. The most important phenolic components of EVOO belong to the chemical classes of lignans, phenolic acids, flavones, and secoiridoids (SIDs): the latter are found only within the family of the Oleaceae and they are considered the main compounds of the phenolic fraction [11]. SIDs are usually glycosidically bound to sugars and produced from the secondary metabolism of terpenes. The most abundant compounds belonging to this family are the dialdehydic forms of elenolic acid linked either to hydroxytyrosol (3,4-DHPE-EDA) or to tyrosol (*p*-HPEA-EDA), an isomer of the oleuropein aglycon (3,4-DHPEA-EA) and a hydroxytyrosol derivative, hydroxytyrosol acetate (3,4-DHPEA-AC) [12,13]. The EVOO SIDs in aglyconic forms arise from glycosides in olive fruits by hydrolysis of endogenous β-glucosidases during crushing and malaxation processes. Lignans are another important family of compounds detected in EVOO [6]. According to Owen and coworkers [14], the quantity of lignans in EVOO may reach 100 mg/kg but, as with the simple phenols and SIDs, there is considerable variation between EVOOs. In particular, as suggested by Bajoub and coworkers [15], the amount of lignans may be used as a varietal marker because several cultivars possess a widely different lignan content within the phenol fraction. For these reasons, the phenolic content of EVOO is highly variable. Overall, EVOO phenolic content is influenced by a range of factors, including: cultivar, pedo-climatic conditions, organic or non-organic cultivation, level of maturation of the drupe at harvest time (early or late harvest), and technology employed in the oil production process. For example, following foliar fertilization no effects were found on either general physico-chemical characteristics or fatty acid composition but a significant decrease in both polyphenol and *o*-diphenol contents was detected [16]. There is a wealth of studies on the influence of irrigation, cultivar, and soil properties on the nutraceutical components and organoleptic properties of EVOO, particularly as regards tocopherols, phenolic fraction, oleic and linoleic acid contents, stability, and sensorial characteristics [5,17]. Olives reach their full size in the fall but may not fully ripen from green to black until late winter. Green olives have slightly less oil, more bitterness, and can be higher in polyphenols [18]. Many people like the peppery and bitter quality of early harvest oil. Flavor notes of grass, green, green leaf, pungent, astringent, are used to describe early harvest fall oils [19]. Early harvest oils often have a longer shelf life due to their higher polyphenol and antioxidant content: hence, these EVOOs are sometimes blended with late harvest EVOOs to improve the latter’s shelf life. Although the phenolic and α-tocopherol content of EVOO are important parameters to be considered, the bioavailability of these compounds is closely related to their structure and is crucial in order to explain the health effects ascribed to them [2]. Another important consideration with regard to the healthful properties of α-tocopherol and phenolics in EVOO is their stability in storage. In fact, the content of phenolic compounds is an important parameter in the evaluation of EVOO quality because phenols greatly contribute to oil flavor (bitterness and pungency) as well as protecting it (together with α-tocopherol) from oxidation [2,18]. Maximum EVOO storage time, although controversial, is generally deemed to be 12–18 months [20]. Indeed, some studies have shown that EVOO phenolic concentration remains relatively stable over this period when the oil is kept under appropriate storage conditions that limit excessive phenolic and fatty acid degradation (i.e., low temperature, light-protected, dry environment) [2]. Hrncirik et al. have clearly shown that the oxidation process in EVOO subjected to accelerated storage conditions (60 °C, dark) is representative of the autoxidation process over shelf life [21], and is accompanied by depletion of phenols and α-tocopherol. Such depletions occur primarily in the initial stage of the oxidation process and, therefore, can serve as “early indicators” of the EVOO oxidative status. Furthermore, during EVOO storage, hydrolytic mechanisms leading to the release of simple phenols—such as hydroxytyrosol and tyrosol—from complex phenols like SIDs may be involved [11]. In light of the above, this study aimed to evaluate 10 North Italian mono-and multi-varietal EVOOs, upon early and late olive harvest, in order to estimate: (i) their variability in the phenolic profile and α-tocopherol content at different stages of olive ripeness; and (ii) the possibility of using these compounds as potential cultivar markers. Finally, we also studied the EVOO antioxidant capacity and evaluated its correlation with phenolic and α-tocopherol contents.

## 2. Results and Discussion

This study evaluated the polyphenol content of organic and non-organic mono- and multi-varietal extra virgin olive oils (EVOOs) from early and late harvested olives (Table 1). Olive oil samples were previously subjected to chemical analyses for determination of free fatty acid content, peroxide value, and UV absorption characteristics (K_232_ and K_270_) carried out in accordance with the analytical methods described in European Union Commission Regulation EEC/2568/91 [22]. This allowed us to categorize the samples as EVOO.

Total phenol content, determined by the Folin-Ciocalteu test and expressed as mg of gallic acid equivalents per 100 g of sample fresh weight (FW), resulted to be significantly higher in early samples obtained from organic multi-varietal EVOOs (Early Harvest Organic Multi-Varietal/EHOMV). Among the early mono-varietal EVOOs, Early Harvest Organic Casaliva (EHOC) showed a significantly higher total phenol content with respect to the other mono-varietal ones, namely Early Harvest Frantoio and Early Harvest Casaliva (EHF and EHC, respectively). Between the late harvest EVOOs, instead, Late Harvest Organic Casaliva (LHOC) showed a significantly higher total phenol content with respect to Late Harvest Casaliva (LHC) and Late Harvest Multi-Varietal (LHMV) as well as the total phenol content of Late Harvest Organic Multi-Varietal (LHOMV) was significantly higher with respect to LHC. Interestingly, the lower total phenol content was measured in the late harvest multi-varietal EVOOs; while this was not seen in late harvest mono-varietal EVOOs (Table 2). This could be due to the significantly different degrees of ripeness of the different varieties analyzed (Table 3).

In fact, total phenol content increased progressively as olives ripened until peaking, after which there was a significant reduction [23]. The ORAC values of EVOOs analyzed in the present study fall within the USDA database range and confirm the same pattern seen in the Folin-Ciocalteu test (Table 2) [24]. In fact, the ORAC value of EHOMV was significantly higher with respect to all early harvest EVOOs (EHF, EHC, EOHC, and Early Harvest Multi-Varietal/EHMV) as well as EHMV resulted significantly higher with respect to all early harvest mono-varietal EVOOs (EHF, EHC, and EOHC). Among the early mono-varietal EVOOs, EHOC showed a significantly higher ORAC value with respect to the other mono-varietal ones (EHF, EHC). Between the late harvest EVOOs, Late Harvest Frantoio (LHF) showed a significantly higher ORAC value with respect to all late harvest EVOOs, as well as an ORAC value of LHMV that was significantly higher with respect to other late harvest mono-varietal EVOOs (LHC and LHOC).

**Figure 1:**
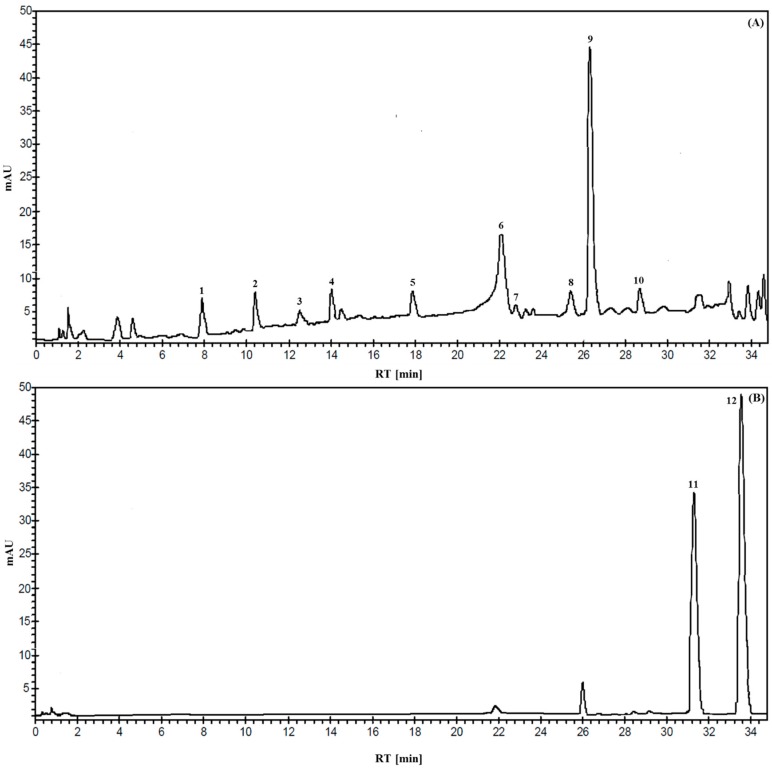
Representative HPLC-DAD chromatogram of Extra-Virgin Olive Oil (EVOO) methanol extract acquired at 280 nm (**A**) and 339 nm (**B**).

Table 4 and Table 5 show the concentration of the identified phenolic compounds in early and late harvest organic and non-organic mono- and multi-varietal EVOOs, confirmed by full-scan MS analysis in negative acquisition mode. Figure 1 shows an exemplificative HPLC-DAD chromatogram of methanol-extracted EVOO acquired at 280 and 330 nm, respectively. The first part of the chromatogram (see box A), is characterized by the presence of a series of simple phenols, i.e., hydroxytyrosol (peak 1), tyrosol (2), vanillic acid (3), vanillin (4). These compounds were widely described in literature [6,25,26]. The second part of the chromatogram contains numerous peaks corresponding to phenols with higher molecular weight, i.e., 3,4-DHPEA-AC (5), 3,4-DHPEA-EDA (6), *p*-HPEA-EDA (7), oleuropein (8), lignans (9), and 3,4-DHPEA–EA (10). Box B shows the only two flavonoids found in the methanol extract, luteolin (11) and apigenin (12). The full-scan MS analysis confirmed the presence of all compounds identified previously, in particular as regards those for which a standard is not commercially available, in line with results obtained by Antonini and coworkers [27].

According to total phenol content and ORAC values, the total polyphenols content, analyzed by HPLC-DAD-MS, resulted in significantly higher EHOMV with respect to all early harvest EVOOs (EHF, EHC, EOHC, and EHMV) as well as EHMV resulted significantly higher with respect to all early harvest mono-varietal EVOOs (EHF, EHC, and EOHC) (Table 4). Furthermore, between early harvest mono-varietal EVOOs, EHOC showed a significantly higher polyphenols content (Table 4).

Among the late harvest EVOOs, instead, LHOMV showed significantly higher total polyphenols content with respect to all late harvest EVOOs (LHF, LHC, LHOC, and LHMV) as well as LHMV resulted significantly higher with respect to all late harvest mono-varietal EVOOs (LHF, LHC, and LHOC) (Table 5). In addition, between late harvest mono-varietal EVOOs, LHOC showed significantly higher polyphenols content (Table 5).

As shown in Table 4 and Table 5, among the polyphenols identified, hydroxytyrosol and tyrosol were determined at low concentrations, while oleuropein metabolites were the most abundant compounds both in early and in late harvest EVOOs. Hydroxytyrosol and tyrosol content was found to be higher in early harvest multi-varietal EVOOs, with the organic being the richest. The lowest hydroxytyrosol and tyrosol contents were found respectively in mono-cultivar Casaliva and organic mono-cultivar Casaliva EVOOs. In the late harvest EVOOs the highest tyrosol and hydroxytyrosol content was found in the organic mono-cultivar Casaliva EVOO, while the lowest was seen in multi-varietal EVOOs. Tyrosol content increases over ripening, and only in the two multi-varietal EVOOs under investigation was it possible to observe a reduction in tyrosol content. It has been widely shown that SIDs and their derivatives have potent antioxidant activity [28]. SIDs represent the most abundant phenolic compounds identified in all early-harvested EVOOs studied, except for EHC, followed by lignans and flavonoids. Otherwise, lignans represent the most abundant phenolic compounds identified in all late-harvested EVOOs studied, except for LHOC, followed by SIDs and flavonoids. Among the SIDs, 3,4-DHPEA-EDA was the most abundant compound. The highest content of the latter was found in early harvest organic mono-cultivar Casaliva (EHOC) sample (108.21 mg/kg), while the lowest concentration was detected in late harvest organic multi-varietal (LHOMV) sample (89.40 mg/kg). The significant presence of SIDs in the phenolic fraction confer potent antioxidant properties on the EVOO samples, which also contributes to extending EVOO shelf-life [28].

During the ripening process, the concentration of phenolic compounds such as 3,4-DHPEA-EDA and *p*-HPEA-EDA increases progressively until it reaches a maximum, after which it decreases [18]. The main environmental factors that probably contribute to the reduction of these compounds are temperature, soil, and water [5]. In fact, rain produces an increase of water content in the drupes. Since polyphenols are soluble both in water and in oil, significant amounts of these compounds are removed during the EVOO production process, due to the fact that these two phases are immiscible [1]. In addition to SIDs, polyphenols most abundantly present in the analyzed EVOOs were lignans: a maximum concentration of 130.99 and 141.46 mg/kg was detected in the early and late harvest organic multi-varietal EVOOs, respectively. The content of these compounds in the examined EVOOs showed an increase during ripening. Accordingly to our data, it is well known that EVOOs generally contain high amounts of lignans, while they have lower levels of some secoiridoid compounds, especially oleuropein aglycon (3,4-DHPEA-EA) [29]. During maturation, chemical and enzymatic activity causes oleuropein to be steadily reduced until it reaches its minimum concentration in ripe olives. Oleuropein is replaced by demethyl-oleuropein and hydroxytyrosol but unrelated to oleuropein concentration [30]. However, during drupe maturation simple phenol content sometimes also decreases due to oxidation and polymerization reactions [30]. Alagna and coworkers identified the main genetic determinants of olive fruit phenolic compounds when evaluating their evolution [31]. A strong correlation emerged between the content of specific metabolites during fruit development and the expression of transcripts involved in their biosynthesis, indicating that the olive polyphenol profile is tightly regulated at a transcriptional level and varies in relation to olive genotype [32]. From this point of view, our data are consistent with data from some Turkish and Tunisian olive varieties [33]. As shown in Table 4, in the early harvest EVOOs the luteolin content is higher than the apigenin one in all cultivars evaluated. In contrast, as shown in the Table 5, the apigenin content is higher than the luteolin one for all cultivars of late harvest EVOOs.

The α-tocopherol content of EVOOs, reported in Table 6, ranged from 29.39 mg/kg in late harvest mono-cultivar frantoio (LHF) sample to 280.67 mg/kg in EHOC sample.

These results are largely in accordance with data previously reported for EVOOs [34], although it is known that altitude and cold temperatures could affect the α-tocopherol content [35]. Early harvest EVOOs were found to possess the highest α-tocopherol content, confirming that α-tocopherol content decreases during olive ripening. Particularly among the early harvest EVOOs, EHOC showed a significantly higher α-tocopherol content with respect to its corresponding non-organic cultivar (EHC) and with respect to EHMV. Among the late harvest EVOOs, LHF showed significantly lower α-tocopherol content with respect to all other ones as well as LHOC and LHMV showing a significantly lower α-tocopherol content with respect to LHC. Furthermore, the LHOC showed a significantly higher α-tocopherol content with respect to LHMV. Finally, the highest variability in α-tocopherol content was shown by the mono-cultivar Frantoio, decreasing from 252 to 29 mg/kg as ripening progressed with respect to the mono-cultivar Casaliva that showed a reduction of less than 27%.

The most innovative aspect of our study is, however, to have analyzed for the first time the content of polyphenols and α-tocopherol and the antioxidant capacity in early and late harvest organic and non-organic, mono and multi-varietal EVOOs coming from the same small geographical origin, the area surrounding the town of Rovereto known as Vallagarina valley in the south-east of the Trentino-Alto Adige region in Italy.

**Figure 2:**
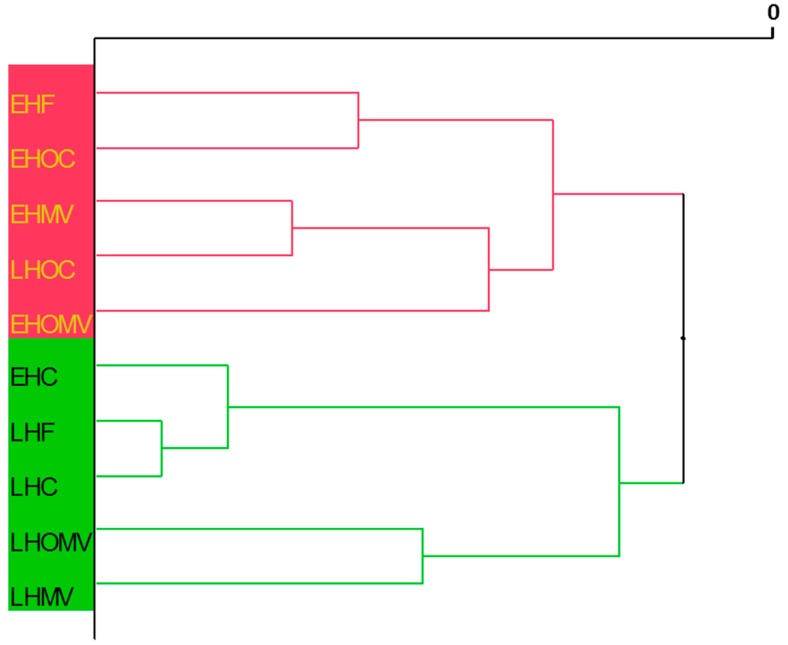
Dendrogram of early and late harvest mono- and multi-varietal Extra-Virgin Olive Oils (EVOOs) obtained from hierarchical cluster analysis (complete linkage). For the meaning of each acronym, refer to Table 1.

In order to elucidate the differences between the studied cultivars and the degree of ripeness, all parameters investigated were subjected to a classification test using a hierarchical cluster analysis. The cluster analysis depicted as a dendrogram in Figure 2 confirms that the analyzed EVOOs are divided into two major classes, early and late harvest EVOOs, with the early harvest samples having the best nutritional and health properties as previously discussed and largely confirmed by the literature [12,17,18,31,33].

Interestingly, within the hierarchical cluster analysis the mono-cultivar Casaliva, there appeared the only EVOO to behave with peculiar characteristics. Indeed, among the studied EVOOs, the relative abundance of the various polyphenols vary, especially with regard to the SIDs fraction, despite the variability in the total polyphenol content being very similar. Overall, SID fraction varied during ripening with a prominent decrease in certain cultivars. The same does not occur for the organic mono-cultivar Casaliva (early vs. late harvest) that shows only a decrease of 15.27% during maturation. This seemed to be a peculiar feature of this cultivar as even the same mono-cultivar grown with conventional farming showed only a slight reduction of the SIDs fraction (13.12%).

Another aspect to consider is that the organic mono-cultivar Casaliva (early vs. late harvest) showed, during ripening, a total phenol content and antioxidant activity very similar (Table 2). This could be explained by the organic farming that is known to positively influence the content of total phenols in the olives as a response to biotic and abiotic stressors while seems to no influence other parameters such as the content of α-tocopherol, as confirmed by our data and others reported in literature [16]. Furthermore, the positive influence of the organic farming is endorsed by the calculated maturity index shown in Table 3. The mono-cultivar Casaliva showed the propensity to a slower ripening compared to the other cultivars in the early harvest time, although starting from a significantly higher maturity index (Table 3). Anyway, the most interesting aspect is that the maturity index of the late harvest organic Casaliva sample (LHOC) which undergoes an increase much lower than the other samples resulting in a significantly lower maturity with respect to all other analyzed late harvest EVOOs. Hence, the hypothesis that the Casaliva cultivar, organically grown, led to an over-production of polyphenols and that, if treated properly by earlier fruit harvesting and rapid milling, may be possible to obtain from it a superior quality of EVOO due to a better control of the influence of both biotic and abiotic stressors [35].

Despite few studies having been carried out to determine the influence of the organic farming on the EVOOs composition, our data suggests that EVOO obtained with organic farming had a higher oxidation stability, preserving its α-tocopherol and total polyphenols content over time. However, these differences are in some cases very small and not statistically significant, especially when considering these parameters individually, thus arriving at inconsistent results depending probably also upon the large season- and genotype-dependent variabilities [35]. Therefore, the authors believe that further large-scale studies on early and late harvest organic and non-organic EVOOs of different geographical origin, cultivar, and genotype are strongly recommended as well as an appropriate cluster analysis that takes into account all sources of variability.

## 3. Materials and Methods

### 3.1. Chemicals

Methanol, *n*-hexane, acetic acid, formic acid, *n-*heptane, tetrahydrofuran, and acetonitrile were HPLC-grade and were purchased from Merck (Darmstadt, Germany). Reference compounds including hydroxytyrosol, tyrosol, vanillin, apigenin, luteolin, oleuropein, gallic acid, vanillic acid, and α-tocopherol were purchased from Sigma-Aldrich (Chemie Gmbh, Steinhein, Germany). 6-Hydroxy-2,5,7,8-tetramethylchromane-2-carboxylic acid (Trolox), Folin-Ciocalteu reagent and sodium carbonate were purchased from Carlo Erba (Milan, Italy); 2,2′-Azobis(2-methylpropionamidine) dihydrochloride (AAPH) was purchased from Fluka (Milan, Italy). Other chemicals were of analytical grade.

### 3.2. Extra-Virgin Olive Oil (EVOO) Samples

EVOOs were selected (see Table 1) and provided anonymously to the laboratory by the scientific responsible of the Rovereto Council, a town in the Vallagarina valley (Trentino-Alto Adige region, Northern part of Italy), as freshly produced early and late harvest organic (according to Article 29(01) of Regulation (EC) No 834/07 and to Article 68 of Regulation (EC) No 889/08) and non-organic mono- (Frantoio and Casaliva cultivars) and multi-varietal olive oils. Selected EVOOs (six samples for each typology, overall 60 samples) were immediately shipped after cold pressing. Upon delivery, EVOOs were aliquoted and stored in the dark and away from heat sources until analysis.

### 3.3. Maturation Index

The Maturation Index (MI) was established as function of skin and pulp fruit color using the method described by Ciafardini and Zullo [36]. Specifically, a group of 100 olives were separated into different categories according to skin color and pulp from deep green (0) to black, with color in the whole pulp (7). Subsequently, the MI was calculated as follows:

MI = a × 0 + b × 1 + c × 2 + d × 3 + e × 4 + f × 5 + g × 6 + h × 7/100

where a, b, c, d, e, f, g, and h represent the fruit numbers of each category. The analyses were repeated three times. Data were shown in Table 3.

### 3.4. 3,4-DHPEA-EA Synthesis by Oleuropein Hydrolytic Conversion

3,4-DHPEA-EA (methyl-4-(2-(3,4-dihydroxyphenethoxy)-2-oxoethyl)-3-formyl-2-methyl-3,4-dihydro-2*H*-pyran-5-carboxylate) was obtained by enzymatic hydrolysis of oleuropein, as a molecular evolution consequence of the hemiacetal functionality of the aglycon **2**, formed by glycosidic bond cleavage. The lipidic/water interface promotes the rapid rearrangement of the intermediate oleuropeinenol **3** into the final stable biomolecule, the transposed secoiridoid **4**, within 5 min [37].

Thus, endogenous β-glucosidase was added to a solution of 100 mg of oleuropein, previously extracted from olive leaves, in 20 mL of a H_2_O/CHCl_3_ 1:1 mixture, at 40 °C for 6 h. The mixture was evaporated and purified using a Waters XTerra C_18_ column on a Varian HPLC system (H_2_O/MeCN gradient) at a flow of 2 mL/min.

**Scheme 1:**
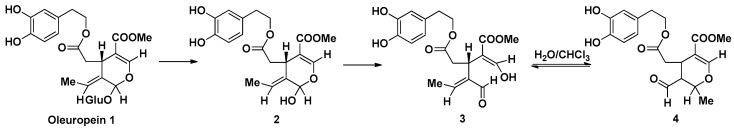
Hydrolytic conversion of Oleuropein to methyl-4-(2-(3,4-dihydroxyphenethoxy)-2-oxoethyl)-3-formyl-2-methyl-3,4-dihydro-2*H*-pyran-5-carboxylate (3,4-DHPEA-EA). The intermediates are described in the Section 3.4 of Materials of Methods.

Methyl-4-(2-(3,4-dihydroxyphenethoxy)-2-oxoethyl)-3-formyl-2-methyl-3,4-dihydro-2*H*-pyran-5-carboxylate was obtained as a yellow oil with 20% yield. ^1^H and ^13^C NMR spectra are in accordance with literature data [38] (Scheme 1).

### 3.5. Sample Preparation

The EVOO phenolic fraction was isolated by liquid–liquid extraction following the procedure devised by Montedoro and coworkers with some modifications, analyzed by HPLC-DAD, and confirmed by HPLC-MS [39]. Briefly, 10 g of EVOO sample was added to 20 mL of methanol/water mixture (8:2 *v*/*v*) five times. The fractions were collected and concentrated under vacuum under a gentle stream of nitrogen at <35 °C until obtaining syrupy consistency. 10 mL of acetonitrile was added to this extract and washed three times with 10 mL of hexane in order to remove the lipid fraction. The sample was then brought to dryness under a gentle stream of nitrogen. Before injection, the phenolic extract was solubilized with 10 mL of methanol and filtered through a PTFE syringe filter 0.2 µm.

### 3.6. Determination of Polyphenol Compounds by HPLC-DAD

HPLC analysis was performed in accordance with Benito and coworkers with some modifications using an Agilent HP1100 system (Agilent Ltd., West Lothian, UK) coupled to a photodiode array detector [23]. The column was an Ascentis 150 × 4.6 mm, 5 µm (Phenomenex, Macclesfield, UK). The elution gradient consisted of mobile phase (A) H_2_O (0.2% CH_3_COOH, pH 3.1) and (B) CH_3_OH. The gradient used was the following: 0–2 min, 95% A and 5% B; 10 min, 75% A and 25% B; 20 min, 60% A and 40% B; 30 min 50% A and 50% B; 40 min, 100% B; 45 min, 100% B. Initial conditions were reached in 15 min with a total run time of 60 min The flow rate was 1.5 mL/min, the injection volume was 20 µL and the column was maintained at 25 °C. Individual phenols were recognized at 280 and 339 nm on the basis of the standards obtained from commercial suppliers described above by comparing their retention time, UV spectra, and mass data. 4-(acetoxyethyl)-1,2-dihydroxybenzene (3,4-DHPEA-AC), 3,4-DHPEA-EDA, *p*-HPEA-EDA and lignans were evaluated using tyrosol as a reference compound. Results are expressed as mg tyrosol equivalents per kg of sample fresh weight (FW).

### 3.7. Confirmation with MS

LC–MS/MS analysis was carried out using a Thermo Scientific UHPLC instrument coupled to a TSQ Quantum Vantage (Thermo Fischer Scientific, San José, CA, USA) triple-stage quadrupole mass spectrometer. Chromatographic separation was achieved using a C18 reversed-phase analytical column, Kinetex C_18_ (2.1 × 50 mm, 1.7 μm particle size, 100 Å, Phenomenex, Torrance, CA, USA). The elution gradient consisted of mobile phase (A) H_2_O (0.1% HCOOH) and (B) CH_3_OH. The gradient used was the following: at *t* = 0.0 min, 90% A and 10% B; at *t* = 1 min, 90% A and 10% B; at *t* = 6 min, 65% A and 35% B; at *t* = 9 min, 65% A and 35% B; at *t* = 11 min, 5% A and 95% B; at *t* = 13 min, 5% A and 95% B; at *t* = 14 min, 90% A and 10% B; at *t* = 15 min, 90% A and 10% B. The flow rate was set at 0.3 mL/min, and the sample injection volume was 10 μL. Mass spectrometric analysis was performed using a heated electrospray ionization (HESI II) source operating in negative ion mode. The following operating conditions were applied: spray voltage, 4.0 kV; vaporizer and capillary temperatures, 280 and 280 °C, respectively; sheath and auxiliary gas at 60 and 20 arbitrary units (au), respectively; S-lens rf amplitude was fixed at 165 V.

Instrument control and data processing was carried out using Xcalibur software v2.0.0 (Thermo Fischer Scientific, San José, CA, USA). The total LC–MS/MS method runtime was 16 min.

### 3.8. α-Tocopherol Evaluation by HPLC-FLU

EVOO samples were prepared and analyzed using the method UNI EN ISO 9936:2011 [40]. Briefly, 100 milligrams of EVOO sample were accurately weighed in a 10 mL volumetric flask and brought to volume with *n*-heptane. The sample was then filtered through a 0.22 μm and injected into the HPLC system. The device, a Shimadzu modular system, comprised a binary pump (LC-30AD), an autosampler (SIL30AC), a fluorescence detector (Model RF 20A) and a thermostatic column oven (Model CTO20AC). The separation was performed on a column LiChrosorb SI-60 (250 × 4.6 mm, 5 µM) maintained at 25 °C, using a mobile phase consisting of a n-heptane/tetrahydrofurane mixture (96.15/3.85, *v*/*v*). The flow rate was 1.0 mL/min and the fluorescence detector was set at the wavelengths of excitation and emission of 295 and 330 nm, respectively. α-tocopherol was quantified by a calibration curve obtained by running the commercially available standard. Results are expressed as mg equivalents of α-tocopherol/kg of sample FW.

### 3.9. Antioxidant Activity Determination

#### 3.9.1. Total Phenols

Total phenol content of EVOO samples was determined colorimetrically using an UV-VIS Spectrophotometer (Shimadzu UV-1601), by the Folin-Ciocalteu method according to Barreca and coworkers [41]. Gallic acid was used as reference compound. Total phenol content is expressed as mg of gallic acid equivalents (GAE)/100 g of sample FW.

#### 3.9.2. Oxygen Radical Absorbance Capacity (ORAC Assay)

Antioxidant activity of sample against 2,2′-azobis(2-amidinopropane)-dihydrochloride (AAPH) peroxyl radical was chemically examined using the ORAC method following Dàvalos and coworkers with some modifications [42]. Briefly, 20 µL of sample solution, diluted in 75 mM phosphate buffer solution pH 7.4, was mixed with 120 µL of fluorescein fresh daily solution (117 nM). After a pre-incubation time of 15 min at 37 °C, 60 µL of freshly prepared AAPH solution (40 mM) was rapidly added. Fluorescence was recorded every 30 seconds for 90 min (λ_ex_ 485; λ_em_ 520) using a Fluorescence Plate Reader (FLUOStar Omega, BMG LABTECH, Ortenberg, Germany) and the decrease in fluorescence was monitored. A blank using phosphate buffer instead of sample and calibration solutions of Trolox (12.5–50 μM) were also included in each assay. The ORAC value was calculated using the area under the fluorescence decay curves and is expressed as µmoles of TE/100 g of sample FW.

## 4. Statistical Analysis

Analytical data were processed using the statistical JMP7 for SAS software (SAS Institute, Cary, NC, USA). All analyses were performed in triplicate (*n* = 3). Data were recorded as mean ± standard deviation (SD). Cluster analysis was performed, using all the variables studied, through multivariate analysis using ward method.

## 5. Conclusions

This is the first study to evaluate both the polyphenols and α-tocopherol content along with the antioxidant capacity of some early and late harvest organic and non-organic mono- and multi-varietal Italian EVOOs coming from a small, well defined area, the Vallagarina valley in Trentino Alto-Adige region, Italy. The cluster analysis showed that the analyzed EVOOs are divided into two major classes, early harvest and late harvest EVOOs, with the early harvest samples which possess the best nutritional and health properties. In light of this, the best behavior was found for the mono-cultivar Casaliva and particularly for the organic sample, which shows only a slight modification of the SIDs fraction (−15.27%) as well as of the total phenol content (+2.04%) and antioxidant activity during ripening (−9%). These results could be explained by organic farming which is known to positively influence the content of total phenols in the olive as a response to biotic and abiotic stressors. Furthermore, this hypothesis was confirmed by the maturity index of LHOC, which showed an increase much lower than other samples, supporting the hypothesis that this cultivar, organically grown, shows an over-production of polyphenols.

## Figures and Tables

**Table 1:** List of the investigated Extra Virgin Olive Oils (EVOOs).

EVOOs	Acronyms	
	Early Harvest	Late Harvest
Frantoio *	EHF	LHF
Casaliva *	EHC	LHC
Organic Casaliva *	EHOC	LHOC
Multi-varietal	EHMV	LHMV
Organic Multi-varietal	EHOMV	LHOMV

* mono-varietal EVOOs.

**Table 2:** Antioxidant properties of early and late harvest mono- and multi-varietal Extra Virgin Olive Oils (EVOOs) methanol extracts. For the meaning of each acronym, refer to Table 1.

EVOOs	Folin Test (mg GAE ^Ψ^/100 g FW *)	ORAC Test (µmol TE ^¥^/100 g FW *)
EHF	21.69 ± 0.46 *^,&^	536.90 ± 1.7153
EHC	20.00 ± 0.53 *^,&^	797.87 ± 5.25 **
EHOC	36.36 ± 1.00 ^&^	1285.97 ± 5.51 **^,b^
EHMV	34.10 ± 1.04 ^&^	1387.37 ± 2.83 **^,b,c^
EHOMV	47.31 ± 0.56	1790.03 ± 2.72 **^,b,c,d^
LHF	34.67 ± 1.94	1250.44 ± 4.54
LHC	25.26 ± 0.58 ^§,$^	1145.13 ± 0.75 ^£^
LHOC	37.10 ± 0.74	1170.15 ± 9.10 ^£^
LHMV	30.63 ^a^ ± 1.10	1060.76 ± 4.72 ^£,#,@^
LHOMV	33.06 ± 1.16	926.95 ± 1.33 ^£,@,γ^

**^Ψ^** GAE = Gallic acid equivalents; * FW = Fresh weight; **^¥^** TE = Trolox equivalents. * *p* < 0.001 vs. EHOC; ^&^ *p* < 0.001 vs. EHOMV; ^§^ *p* < 0.001 vs. LHOC; ^$^ *p* < 0.001 vs. LHOMV; ^a^ *p* = 0.001 vs. LHOC. ** *p* < 0.001 vs. EHF; ^b^ *p* < 0.001 vs. EHC; ^c^ *p* < 0.001 vs. EHOC; ^d^ *p* < 0.001 vs. EHMV; ^£^ *p* < 0.001 vs. LHF; ^#^ *p* < 0.001 vs. LHC; ^@^ *p* < 0.001 vs. LHOC; ^γ^ *p* < 0.001 vs. LHMV.

**Table 3:** Maturation index (M.I.) of the investigated Extra-Virgin Olive Oils (EVOOs). For the meaning of each acronym, refer to Table 1.

EVOOs	M.I.
EHF	1.92 ± 0.03
EHC	2.26 ± 0.02 *
EHOC	1.45 ± 0.02 *^,$^
EHMV	1.54 ± 0.04 *^,$^
EHOMV	1.32 ± 0.01 *^,$,&,§^
LHF	3.58 ± 0.05
LHC	3.87 ± 0.15
LHOC	2.78 ± 0.08 ^£^
LHMV	3.93 ± 0.12 ^a^
LHOMV	3.86 ± 0.06 ^a^

* *p* < 0.001 vs. EHF; ^$^ *p* < 0.001 vs. EHC; ^&^ *p* < 0.001 vs. EHOC; ^§^ *p* < 0.001 vs. EHMV; ^£^ *p* < 0.001 vs. LHC; ^a^ *p* < 0.001 vs. LHOC.

**Table 4:** Phenolic profile of the early harvest mono- and multi-varietal Extra-Virgin Olive Oils (EVOOs). For the meaning of each acronym, refer to Table 1.

Phenolic Compounds	EHF	EHC	EHOC	EHMV	EHOMV
	(mg/kg)				
Hydroxytyrosol	0.89 ± 0.00	0.71 ± 0.67	2.32 ± 0.12	6.35 ± 0.22	11.44 ± 0.55
Tyrosol	4.20 ± 0.64	3.13± 0.41	1.71 ± 0.07	2.54 ± 0.39	5.59 ± 0.56
Vanillic acid	1.80 ± 0.15	2.29 ± 0.02	0.90 ± 0.06	2.01 ± 0.37	1.42 ± 0.10
Vanillin	3.28 ± 0.06	3.44 ± 0.27	4.26 ± 0.48	4.99 ± 0.41	4.84 ± 0.18
3,4-DHPEA-AC	1.25 ± 0.16	1.78 ± 0.18	1.67 ± 0.25	3.43 ± 0.20	1.72 ± 0.12
3,4-DHPEA-EDA	76.67 ± 5.83	56.57 ± 4.73	108.21 ± 9.56	102.44 ± 4.63	106.89 ± 12.58
*p*-HPEA-EDA	6.65 ± 0.97	7.42 ± 0.44	12.29 ± 1.22	10.98 ± 1.01	12.46 ± 0.62
Oleuropein	18.51 ± 1.61	6.61 ± 0.66	12.85 ± 1.91	10.01 ± 1.11	15.41 ± 1.47
Lignans	69.20 ± 1.77	79.90 ± 7.73	83.81 ± 1.91	111.17 ± 5.22	130.99 ± 9.72
3,4-DHPEA-EA	7.98 ± 0.43	3.18 ± 0.12	9.18 ± 0.97	5.50 ± 0.12	8.84 ± 2.96
Luteolin	13.25 ± 1.98	4.67 ± 0.45	22.54 ± 2.91	11.76 ± 0.95	17.72 ± 1.26
Apigenin	5.47 ± 0.56	4.83 ± 0.24	3.39 ± 0.57	8.43 ± 1.24	8.87 ± 1.48
Total	209.15	174.53 *^,$,§^	263.13 *	280.16 *^,$^	326.19 *^,$,§,&^

* *p* < 0.001 vs. EHF; ^$^ *p* < 0.001 vs. EHOC; **^§^** *p* < 0.001 vs. EHMV; ^&^ *p* < 0.001 vs. EHC.

**Table 5:** Phenolic profile of the late harvest mono- and multi-varietal Extra-Virgin Olive Oils (EVOOs). For the meaning of each acronym, refer to Table 1.

Phenolic Compounds	LHF	LHC	LHOC	LHMV	LHOMV
	(mg/kg)				
Hydroxytyrosol	3.04 ± 0.16	1.99 ± 0.31	4.29 ± 0.34	0.49 ± 0.03	0.58 ± 0.04
Tyrosol	4.26 ± 0.33	4.56 ± 0.44	4.93 ± 0.80	1.12 ± 0.13	2.84 ± 0.36
Vanillic acid	2.79 ± 0.18	3.34 ± 0.09	1.42 ± 0.14	1.13 ± 0.16	1.38 ± 0.12
Vanillin	3.07 ± 0.34	3.55 ± 0.20	3.75 ± 0.10	0.53 ± 0.02	2.64 ± 0.14
3,4-DHPEA-AC	1.96 ± 0.23	1.50 ± 0.19	3.94 ± 0.23	2.98 ± 0.18	1.95 ± 0.25
3,4-DHPEA-EDA	62.17 ± 4.48	55.04 ±0.79	98.92 ± 13.33	54.60 ± 3.00	89.41 ± 1.29
*p*-HPEA-EDA	2.23 ± 0.08	2.65 ± 0.23	7.32 ± 0.10	0.88 ± 0.02	14.38 ± 1.27
Oleuropein	5.28 ± 0.25	1.80 ± 0.13	2.70 ± 0.24	1.52 ± 0.13	9.53 ± 0.67
Lignans	100.00 ± 22.05	92.29 ± 6.14	106.00 ± 7.42	176.77 ± 14.41	141.96 ±6.42
3,4-DHPEA-EA	2.55 ± 0.27	1.44 ± 0.04	3.49 ± 0.12	6.37 ± 0.59	2.24 ± 0.14
Luteolin	2.03 ± 0.15	0.78 ± 0.04	1.75 ± 0.11	7.36 ± 0.82	10.70 ± 0.31
Apigenin	3.89 ± 0.31	3.5 ± 0.272	3.37 ± 0.10	13.62 ± 1.79	14.73 ± 0.80
Total	193.27	172.44	241.88 **	267.67 ^§^	292.34 *^,&^

* *p* = 0.001 vs. LHF; ^&^ *p* < 0.001 vs. LHC; ^§^ *p* < 0.001 vs. LHF; ** *p* < 0.001 vs. LHC.

**Table 6:** α-Tocopherol content of early and late harvest mono- and multi-varietal Extra-Virgin Olive Oils (EVOOs). For the meaning of each acronym, refer to Table 1.

EVOOs	α-Tocopherol (mg/kg)
EHF	252.65 ± 18.24
EHC	170.62 ± 13.20 *
EHOC	280.67 ± 6.28 **
EHMV	198.39 ± 10.27
EHOMV	192.73 ± 15.51
LHF	29.39 ± 1.94
LHC	125.51 ± 11.35 ^&^
LHOC	62.19 ± 1.62 ^&,$^
LHMV	46.68 ± 2.72 ^&,$,§^
LHOMV	70.57 ± 6.37 ^&^

* *p* < 0.001 vs. EHOC; ** *p* < 0.001 vs. EHMV; ^&^ *p* < 0.001 vs. LHF; ^$^ *p* < 0.001 vs. LHC; ^§^ *p* = 0.001 vs. LHOC.
